# Molecular alterations in *IDH*-mutant astrocytoma: A multi-institutional retrospective study

**DOI:** 10.1093/noajnl/vdaf088

**Published:** 2025-04-28

**Authors:** Keng Lam, Hanim I Ozkizilkaya, Denái R Milton, Antonio Dono, Yajie Liu, Suprateek Kundu, Vinodh A Kumar, Jason M Johnson, Yoshua Esquenazi, Chirag B Patel, Leomar Y Ballester

**Affiliations:** Department of Neuro-Oncology, The University of Texas MD Anderson Cancer Center, Houston, Texas, USA; Department of Pathology, University of Southern California; Department of Biostatistics, The University of Texas MD Anderson Cancer Center, Texas, USA; Department of Neurosurgery, The University of Texas Health Science Center at Houston, Houston, Texas, USA; Department of Biostatistics, The University of Texas MD Anderson Cancer Center, Texas, USA; Department of Biostatistics, The University of Texas MD Anderson Cancer Center, Texas, USA; Department of Neuroradiology, The University of Texas MD Anderson Cancer, Houston, USA; Radiology and Biomedical Imaging, Yale University, New Haven, USA; Department of Neurosurgery, The University of Texas Health Science Center at Houston, Houston, Texas, USA; Department of Neuro-Oncology, The University of Texas MD Anderson Cancer Center, Houston, Texas, USA; Division of Pathology and Laboratory Medicine, The University of Texas MD Anderson Cancer, Houston, Texas, USA

**Keywords:** astrocytoma, brain tumor, diffuse glioma, IDH1, IDH2, molecular alterations, prognosis, WHO grade

## Abstract

**Background:**

Several molecular alterations have been identified to provide prognosis for patients with isocitrate dehydrogenase (IDH)-mutant astrocytoma. However, contemporary baseline survival data with respect to their molecular alterations are lacking. The prognostic value of histologic grading remains controversial.

**Methods:**

This was a retrospective multi-site study of adult IDH-mutant diffuse astrocytoma patients. Overall survival (OS) was estimated using the Kaplan-Meier method. Associations between OS and measures of interest were evaluated using Cox proportional hazards regression models.

**Results:**

We identified 241 eligible patients. The most frequent mutations were IDH1 (98%), TP53 (91%), ATRX (70%), ARID1A (8%), BRCA2 (6%), TSC2 (6%), CDKN2A (6%), and CREBBP (6%). IDH2 mutations were identified in 2%. By univariate analysis, age > 40 (hazard ratio [HR], 2.03; 95% CI, 1.20-3.45; *p* = .009) was associated with worse OS. Wildtype BRCA2 compared with mutated BRCA2 (HR, 0.42; 95% CI, 0.20-0.90; *p* = .024) and Central Nervous System World Health Organization (CNS WHO) grade 2 astrocytoma compared with grade 3 disease (HR, 0.40; 95% CI, 0.21-0.78; *p* = .007) were associated with better OS. In multivariable analysis, age > 40 (HR, 2.06; 95% CI, 1.18-3.59; *p* = .011) was associated with worse OS and CNS WHO grade 2 (HR, 0.42; 95% CI, 0.21-0.83; *p* = .012) remained associated with improved OS. We identified an association between increased tumor mutation burden (TMB) and worse OS.

**Conclusions:**

Age and CNS WHO grade remain essentials for risk stratification among *IDH*-mutant astrocytoma patients. Further studies are warranted to determine the prognostic implications of *BRCA2* mutations and TMB.

**Key Points**

- In this *IDH*-mutant astrocytoma cohort, age and CNS WHO grade were identified as significant factors for risk stratification.
- *BRCA2* mutations could play a role in risk stratification of *IDH*-mutant astrocytoma.
- TMB may play a role in risk stratification of *IDH*-mutant astrocytomas.

**Importance of the Study**

Having contemporary baseline survival data for isocitrate dehydrogenase (*IDH*)-mutant astrocytomas with respect to molecular alterations is crucial for making informed treatment decisions. Moreover, the prognostic value of CNS WHO grade in *IDH*-mutant astrocytomas remains controversial. We conducted a multi-institutional retrospective study of IDH-mutant astrocytomas. In univariate analysis, age > 40 years was associated with poor overall survival (OS). *BRCA2* wild-type status was associated with better OS in univariate analysis but not in multivariable analysis. Compared with CNS WHO grade 3 astrocytoma, grade 2 disease was associated with better OS. Multivariable analysis confirmed age and grade as prognostic factors. We also identified a correlation between TMB and worse OS. Further studies are warranted to determine the role of *BRCA2* mutation and TMB in the prognosis of *IDH*-mutant astrocytomas.

In the current (5th) edition of the World Health Organization (WHO) Classification of Tumors of the Central Nervous System (CNS), molecular alterations play a critical role in the diagnosis and grading of adult diffuse gliomas.^1^ For astrocytoma with isocitrate dehydrogenase (*IDH*) mutations (*IDH1* or *IDH2*), investigators have attempted to risk-stratify survival based on patients’ molecular alterations.^2^ For the stratification of patients with *IDH*-mutant astrocytomas, the prognostic value of CNS WHO grade, which is primarily based on histologic parameters, remains controversial. Moreover, the use of new brain-penetrant therapies that target the mutant IDH1 and IDH2 enzymes (eg, vorasidenib), is on the rise for the treatment of low-grade *IDH*-mutant astrocytomas.^3^ Hence, accurate risk stratification of patients with *IDH*-mutant astrocytomas is critical in guiding treatment decisions.

Despite the widespread availability of next-generation sequencing, contemporary baseline survival data for patients with *IDH*-mutant astrocytomas with respect to their tumor’s molecular alterations are lacking.^4^*CDKN2A/B* deletion and *MGMT* promoter methylation are known to be prognostic and predictive, respectively, for survival in patients with *IDH*-mutant astrocytomas.^5,6^*MYCN, CDK4,* amplification, and mutations in mismatch repair (MMR) genes have also been postulated as a marker of worse prognosis in patients with *IDH*-mutant astrocytoma.^7–9^ For risk stratification, however, the potential value of other molecular alterations routinely identified on sequencing tests, requires additional studies with large patient cohorts.^10^ Furthermore, the role of WHO grading for *IDH*-mutant astrocytomas, in particular CNS WHO grade 2 and 3 tumors, remains unclear.^11^ We performed the present study to address these questions.

## Materials and Methods

### Patient Cohort

*IDH*-mutant diffuse astrocytoma patients at two different institutions were retrospectively identified. All patients were at least 16 years old, and their tumor samples were collected from 2002 to 2022. Patients with incomplete molecular information or treatment data were excluded. Patients with *1p/19q co-deletion* (oligodendrogliomas) were also excluded. Approval of the study was obtained from the institutional review boards of both institutions prior to collecting and analyzing the data.

### Histological and Molecular Analysis

Board-certified neuropathologists from both institutions reviewed and classified all tumors following the criteria from the 2021 WHO Classification of Tumors of the CNS.^1^ Both institutions identified *IDH1*/*IDH2* mutations, which can be canonical (*IDH1 p.R132H* via immunohistochemistry) or noncanonical (other *IDH1*/*IDH2* alterations via next-generation sequencing [NGS]). Sequencing of all cases was performed in clinical laboratories accredited by the College of American Pathologists and certified by the Clinical Laboratory Improvement Amendments to perform high-complexity testing. Sequencing was performed by a targeted NGS panel consistently interrogating 205 genes and 26 gene rearrangements, as well as genomic signatures including microsatellite instability (MSI) and tumor mutational burden (TMB) (FoundationOne, Foundation Medicine, Inc., Cambridge, MA, USA) as previously described for Institution #2 patients. Patients from Institution #1 were analyzed by a high-throughput NGS assay for the detection of sequence variants/mutations in the coding sequence of 134 genes and selected copy number variations (amplifications) in 47 genes (overlap: 146 genes total) or a custom assay interrogating 610 genes (single nucleotide variants and insertion/deletion alterations), copy number variants in 583 genes, select gene rearrangements in 34 genes, and selected genomic immune-oncology signatures including MSI and TMB.^12^

Ki67 staining (MIB-1 clone, Bio CARE API 3156AA H, ready for use) was performed using a Leica Bond iii Autostainer. The Ki67-stained glass slides were digitized and analyzed with ImageScope using an algorithm to quantify positive nuclei in the area with highest staining and highest cellular density (ie, hot spot—1000–2000 cells were analyzed per area) and over multiple regions (4000–8000 cells were analyzed per specimen). Computer-assisted quantitation of Ki67 labeling was only available for Institution #1 patients.

### Treatments

Patient treatment data were abstracted from electronic medical records. The extent of resection (EOR) was classified as either gross total resection (GTR) or non-GTR based on the postoperative brain imaging reviewed by a board-certified neuroradiologist. All patients at some point underwent radiation therapy with concurrent and/or adjuvant chemotherapy or under imaging surveillance only at the discretion of the treating neuro-oncologist.

### Statistical Analysis

The primary outcome measure for this analysis was overall survival (OS). OS time was computed from the original diagnosis surgery date to the last follow-up visit date or known death date. Patients alive at the date of the last follow-up visit were censored. Progression-free survival (PFS) was computed from collection date/surgery date to date of disease progression or death (if died without disease progression) or the last follow-up date. Patients who were alive and did not experience progression of disease at the last follow-up date were censored. The Kaplan-Meier (K-M) method was used to estimate OS and PFS, and the log-rank test was used to assess differences in OS and PFS between groups. In addition, Cox proportional hazards regression models were used to assess the association between measures of interest and survival. Measures with *p*-values < .10 in the univariate model were included in a multivariable model. The mean TMB between Institution #1 and Institution #2 cohorts was compared using both non-parametric and parametric methods. Non-parametric tests included the Wilcoxon rank-sum test and the permutation test. Additionally, a two-sample *t*-test was performed after normalizing TMB values with log and square-root transformations. *p*-Values < .05 were considered statistically significant. All statistical analyses were performed using SAS software (version 9.4 for Windows) and RStudio software (with R version 4.3.3 for Mac). No adjustments for multiple testing were made.

## Results

### Patient Characteristics

We identified a total of 241 eligible patients, consisting of 185 (77%) patients at Institution #1 and 56 (23%) patients at Institution #2. **Table 1** summarizes the overall patient characteristics. Fifty-nine percent of the patients were male, and the median age was 33 years (range, 16-73 years). Ninety percent of the patients were Caucasian, with a higher proportion of racial and ethnic minorities in Institution #2 patients (20% vs 8% at Institution #1; *p* = 0.021). The majority of patients had CNS WHO grade 2 (41%), followed by grade 4 (31%) and grade 3 (28%) gliomas. Of the 185 patients with EOR data, 74% underwent non-GTR procedures. Most of the patients had canonical *IDH1 p.R132H* mutation (80%). The following genes were mutated in at least 5% of the patients: *IDH1* (98%), *TP53* (91%), *ATRX* (70%), *ARID1A* (8%), *BRCA2* (6%), *TSC2* (6%), *CDKN2A* (6%), and *CREBBP* (6%). *IDH2* mutations were identified in 2% of cases. For *CDKN2A/B*, the reported alterations are copy number loss, but otherwise all other alterations were single nucleotide variants. More details regarding their mutations can also be found under **Supplementary Table S1** and **Supplementary Excel File**.

**Table 1.** Summary of Patient Characteristics

Characteristic	*n* (%)			*p* ^a^
	All Patients (*N* = 241)	Institution #1 Cohort (*N* = 185)	Institution #2 Cohort (*N* = 56)	
Sex				
Male	142 (59)	108 (58)	34 (61)	.88
Female	99 (41)	77 (42)	22 (39)	
Median age, years (range)	33.1 (16.3-73.0)	32.5 (16.3-65.3)	35.5 (20.0-73.0)	**.014** ^b^
Race				
White	216 (90)	171 (92)	45 (80)	**.021**
Other	25 (10)	14 (8)	11 (20)	
CNS WHO Grade				
2	96 (41)	84 (47)	12 (21)	**<.001**
3	66 (28)	51 (28)	15 (27)	
4	74 (31)	45 (25)	29 (52)	
Unknown	5	5	0	
RT as First Line, *N*				
No	68 (29)	61 (34)	7 (13)	**.002**
Yes	169 (71)	120 (66)	49 (88)	
Unknown	4	4	0	
ST as First Line, *N*				
No	80 (34)	70 (39)	10 (18)	**.004**
Yes	156 (66)	110 (61)	46 (82)	
Unknown	5	5	0	
TMZ as First Line, *N*				
No	82 (35)	72 (40)	10 (18)	**.002**
Yes	153 (65)	107 (60)	46 (82)	
Unknown	6	6	0	
PC as First Line, *N*				
No	235 (99)	179 (88)	56 (100)	1.00
Yes	3 (1)	3 (2)	0	
Unknown	3	3	0	
H&E, *N*	28	28	0	
Median (range)	4 (0-18)	4 (0-18)	--	
PHH3, *N*	22	22	0	
Median (range)	6.0 (0-17)	6.0 (0-17)	-	
Initial Ki67—average, *N*	86	86	0	
Median (range)	5.1 (0-51)	5.1 (0-51)	-	
Initial Ki67—hotspot, *N*	68	68	0	
Median (range)	7.0 (0.6-56)	7.0 (0.6-56)	-	
EOR				
Non-GTR	137 (74)	95 (74)	42 (75)	1.00
GTR	48 (26)	34 (26)	14 (25)	
Missing	56	56	0	
*IDH* mutation				
Noncanonical	46 (19)	37 (20)	9 (16)	.67
Canonical	194 (80)	147 (79)	47 (84)	
Both	1 (< 1)	1 (1)	0	
Vital status				
Alive	176 (73)	145 (78)	31 (55)	**.001**
Died	65 (27)	40 (22)	25 (45)	
Median follow-up time, months (range)	45.2 (0.5-246.9)	43.2 (0.5-246.9)	49.2 (2.3-158.4)	.84^b^

^a^Fisher’s exact test or its generalization;

^b^Wilcoxon rank-sum test.

**Abbreviations:** CNS WHO, Central Nervous System World Health Organization; EOR, extend of resection; GTR, gross total resection; H&E, Hematoxylin and eosin staining; PC; procarbazine hydrochloride and lomustine; RT, radiation treatment; ST, systemic treatment; TMZ, temozolomide; WT, wild-type. Bold numbers represent statistically significant values.

Seventy-one percent of patients underwent radiation therapy, and 66% received systemic therapy upfront (almost exclusively temozolomide) as shown in **Table 1**. Twenty-seven percent of the patients died during the study, with a median follow-up time of 45.2 months (range, 0.5-246.9 months). Institution #2 patients were older (median age, 35.5 years vs. 32.5 years at Institution #1; *p* = .014), and a higher percentage of them had a CNS WHO grade 4 glioma (45% vs 17%; *p* < .001). Furthermore, a higher percentage of patients in Institution #2 were dead at the time of the study (45% vs 22%; *p* = .001).

### Univariate Analysis

**Figure 1.**
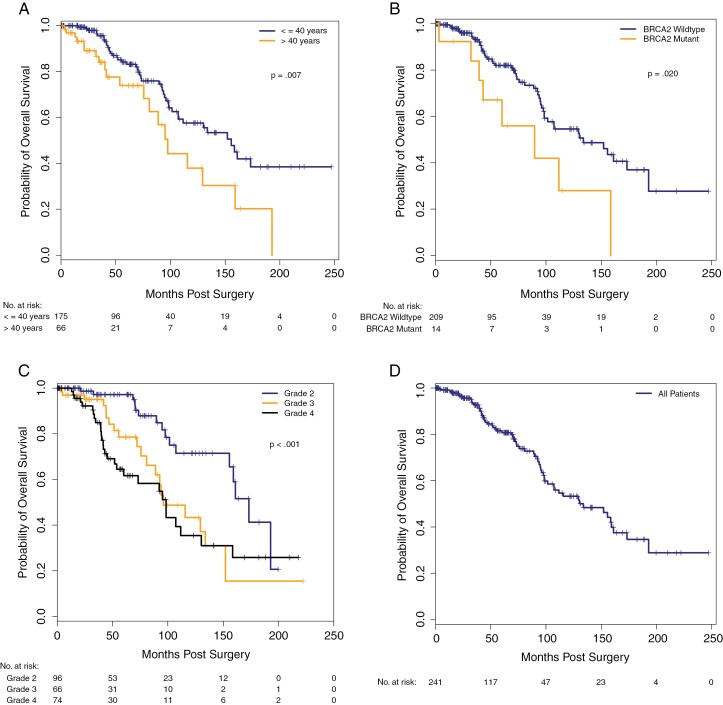
Overall survival—by (A) age (B) *BRCA2* mutation status (C) CNS WHO Grade and (D) all patients

**Table 2** presents univariate analysis results for OS. The median OS for the overall cohort was 133.7 months. For the combined cohort, age >40 years (hazard ratio [HR], 2.03; 95% CI, 1.20-3.45; *p = .009*) was significantly associated with worse OS. Also, wildtype *BRCA2* status was associated with better OS (HR, 0.42; 95% CI, 0.20-0.90; *p = .024*). CNS WHO grade was also significantly associated with OS. Using grade 3 astrocytoma as the reference group, grade 2 disease was associated with significantly better OS (HR, 0.40; 95% CI, 0.21-0.78; *p = .007*). There was no OS difference between the *IDH* canonical and noncanonical groups (HR, 1.24; 95% CI, 0.65-2.38; *p* = .52). **Figure 1** illustrates the K-M plots for OS.

**Table 2.** Summary of Univariate Analysis Results for OS

Characteristic	Median OS Time, Months (95% CI)	OS Rate, % (YR1:YR5:YR10: Last)	*p*	HR (95% CI)	*p*
**All patients**					
Overall	133.7 (98.4-161.0)	99:82:53:29			
Age					
≤40 years	155.5 (107.0-NE)	100:84:58:39	**.007**	*Ref*	
>40 years	97.6 (75.7-159.1)	97:74:38:0		2.03 (1.20-3.45)	**.009**
CNS WHO grade					
2	173.2 (155.5-NE)	100:97:71:21	**<.001**	0.40 (0.21-0.78)	**.007**
3	96.0 (80.8-152.0)	97:79:43:15		*Ref*	
4	98.4 (53.7-130.1)	100:64:35:26		1.21 (0.68-2.16)	.51
EOR					
Non-GTR	98.4 (92.7-152.0)	100:80:43:9	.29	*Ref*	
GTR	159.1 (107.0-NE)	98:82:54:27		0.71 (0.38-1.33)	.29
EOR (HG2)					
Non-GTR	161.0 (101.5-192.8)	100:95:62:0	.77	*Ref*	
GTR	159.1 (107.0-NE)	100:94:67:22		0.85 (0.29-2.51)	.77
EOR (HG3)					
Non-GTR	96.0 (88.9-133.7)	100:87:36:8	.83	*Ref*	
GTR	115.5 (3.1-NE)	89:74:30:30		0.89 (0.29-2.67)	.83
EOR (HG4)					
Non-GTR	51.8 (41.5-60.1)	100:34:23:23	.22	*Ref*	
GTR	NE (32.0-NE)	100:69:69:69		0.50 (0.16-1.55)	.23
*IDH* mutation					
Noncanonical	159.1 (73.1-NE)	100:89:64:38	.52	*Ref*	
Canonical	129.3 (97.6-173.2)	99:79:51:30		1.24 (0.65-2.38)	.52
CDKN2A/B*					
Mutation	98.4 (73.1-158.4)	100:74:34:26	.20	*Ref*	
WT	152.0 (94.8-NE)	99:82:59:44		0.67 (0.36-1.25)	.21
*ARID1A*					
Mutation	173.2 (60.1-NE)	100:87:68:26	.78	*Ref*	
WT	130.1 (95.1-161.0)	99:80:51:36		1.13 (0.48-2.66)	.78
*ATM*					
Mutation	92.7 (4.7-173.2)	90:70:32:0	.14	*Ref*	
WT	130.1 (98.4-161.0)	100:82:53:30		0.56 (0.25-1.23)	.15
*ATRX*					
Mutation	152.0 (96.0-173.2)	99:83:55:35	.56	*Ref*	
WT	98.4 (92.2-NE)	98:74:43:43		1.21 (0.64-2.28)	.56
*BRCA2*					
Mutation	89.7 (32.0-158.4)	92:67:28:0	**.020**	*Ref*	
WT	133.7 (98.4-192.8)	99:82:55:28		0.42 (0.20-0.90)	**.024**
*CDKN2A*					
Mutation	92.2 (32.7-158.4)	100:69:46:0	.19	*Ref*	
WT	130.1 (98.4-192.8)	99:82:52:30		0.59 (0.27-1.31)	.20
*CREBBPA*					
Mutation	152.0 (42.9-173.2)	92:74:63:0	.53	*Ref*	
WT	130.1 (96.0-NE)	99:81:52:38		0.76 (0.32-1.78)	.53
*IDH1*					
Mutation	129.3 (98.4-161.0)	99:81:51:27	.29	*Ref*	
WT	NE (NE-NE)	100:100:100:100		0.44 (0.03-7.39)	.57
*TP53*					
Mutation	129.3 (98.4-161.0)	99:81:51:30	.63	*Ref*	
WT	130.1 (39.4-173.2)	100:81:61:0		1.25 (0.50-3.13)	.63
*TSC2*					
Mutation	107.1 (41.9-173.2)	100:79:38:0	.14	*Ref*	
WT	133.7 (98.4-192.8)	99:81:54:30		0.59 (0.29-1.20)	.14
**Institution #1 cohort**					
Overall	133.7 (98.4-161.0)	99:82:53:29			
Age					
≤40 years	161.0 (130.1-NE)	100:88:65:45	.18	*Ref*	
>40 years	129.3 (80.8-192.8)	98:86:57:0		1.62 (0.79-3.33)	.19
CNS WHO grade					
2	173.2 (155.5-NE)	100:97:74:21	.09	0.58 (0.26-1.29)	.18
3	133.7 (92.7-NE)	98:83:63:22		*Ref*	
4	107.0 (60.1-NE)	100:75:41:34		1.28 (0.58-2.82)	.54
H&E					
Continuous	159.1 (70.5-NE)	100:100:51:17		1.07 (0.93-1.23)	.36
PHH3					
Continuous	161.0 (95.1-NE)	100:92:73:49		0.98 (0.79-1.23)	.89
Initial Ki67 average					
Continuous	161.0 (129.3-NE)	100:91:71:49		1.07 (1.03-1.12)	**.002**
Initial Ki67 hotspot					
Continuous	161.0 (152.0-NE)	100:78:78:39		1.06 (1.02-1.10)	**.001**
EOR					
Non-GTR	129.3 (98.4-161.0)	100:87:54:12	.25	*Ref*	
GTR	159.1 (107.0-NE)	100:92:60:30		0.62 (0.28-1.41)	.26
*IDH* mutation					
Noncanonical	159.1 (70.5-NE)	100:90:66:49	.91	*Ref*	
Canonical	155.5 (111.5-NE)	99:87:62:36		1.05 (0.48-2.29)	.91
CDKN2A/B*					
Mutation	107.0 (40.9-NE)	100:80:36:36	.11	*Ref*	
WT	NE (133.7-NE)	99:92:76:58		0.50 (0.21-1.18)	.12
*ATRX*					
Mutation	173.2 (133.7-NE)	100:93:72:47	.09	*Ref*	
WT	98.4 (94.8-NE)	98:78:44:44		2.01 (0.89-4.54)	.10
*IDH1*					
Mutation	155.5 (115.5-NE)	99:87:61:33	.42	*Ref*	
WT	NE (NE-NE)	100:100:100:100		0.74 (0.04-13.17)	.84
*TP53*					
Mutation	161.0 (115.5-NE)	99:87:61:38	.37	*Ref*	
WT	130.1 (33.4-173.2)	100:90:60:0		1.60 (0.57-4.53)	.37
**Institution #2 cohort**					
Overall	89.7 (53.7-97.6)	98:66:29:0			
Age					
≤ 40 years	92.2 (72.0-158.4)	100:72:39:0	**.040**	*Ref*	
> 40 years	75.7 (32.0-97.6)	94:53:0:0		2.31 (1.01-5.27)	**.046**
CNS WHO grade					
2	NE (89.7-NE)	100:100:63:63	**.038**	0.17 (0.03-0.79)	**.025**
3	75.7 (44.1-93.8)	93:70:0:0		*Ref*	
4	53.7 (41.5-NE)	100:50:33:0		0.87 (0.37-2.07)	.75
EOR					
Non-GTR	89.7 (72.0-97.6)	100:70:30:0	.27	*Ref*	
GTR	NE (14.5-NE)	93:57:57:57		1.77 (0.64-4.95)	.27
*IDH mutation*					
Noncanonical	73.1 (32.0-158.4)	100:86:43:0	.45	*Ref*	
Canonical	89.7 (51.8-97.6)	98:63:27:27		1.74 (0.41-7.43)	.45
CDKN2A/B*					
Mutation	73.1 (32.7-158.4)	100:60:30:0	.88	*Ref*	
WT	89.7 (53.7-97.6)	98:67:28:28		0.92 (0.34-2.49)	.88
*ATRX*					
Mutation	88.9 (53.7-96.0)	98:66:24:0	.41	*Ref*	
WT	NE (41.7-NE)	100:67:50:50		0.64 (0.22-1.87)	.41
*TP53*					
Mutation	89.7 (72.0-97.6)	98:67:27:0	.75	*Ref*	
WT	NE (39.4-NE)	100:50:50:50		0.72 (0.10-5.41)	.75

**Abbreviations:** CNS WHO, Central Nervous System World Health Organization; EOR, extend of resection; GTR, gross total resection; H&E, Hematoxylin and eosin staining; HG2, histological grade 2; HG3, histological grade 3; HG4, histological grade 4; Last, as of last assessment date; NE, not estimated/not reached; *ref*, reference group; WT, wild-type; YR1, year 1 after initial diagnosis; YR10, year 10 after initial diagnosis; YR5, year 5 after initial diagnosis. * = copy number loss; all molecular alterations are otherwise single nucleotide variants.

Similarly, in the Institution #2 cohort, age > 40 years was associated with worse OS compared to age < 40 years (HR, 2.31; 95% CI, 1.01-5.27; *p* = .046), and patients with CNS WHO Grade 2 astrocytoma had better OS than did those with grade 3 disease (HR, 0.17; 95% CI, 0.03-0.79; *p* = .025). Among the Institution #1 patients, increased Ki67 labeling (average Ki67 level: HR, 1.07; 95% CI, 1.03-1.12; *p* = .002 and Ki67 hotspot: HR, 1.06; 95% CI, 1.02-1.10; *p* = .001) was associated with worse OS.

**Figure 2.**
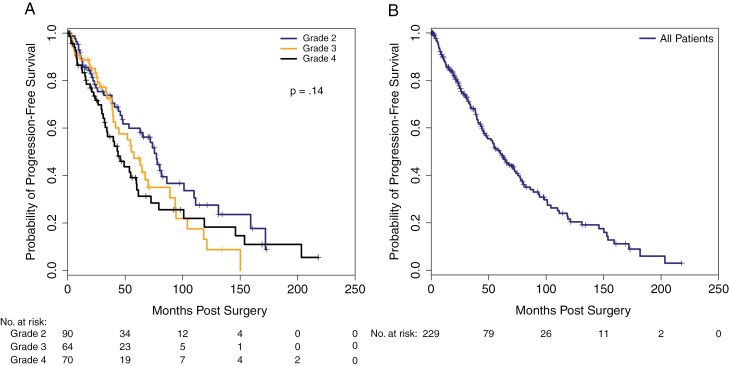
Progression free survival by (A) CNS WHO grade and (B) All patients.

The median PFS for the overall cohort was 59.7 months in which CNS WHO Grade was not associated with worse PFS (*p* = .14) as shown in Figure 2 and Supplementary Table S2.

### Multivariable Analysis

Table 3 presents the multivariable analysis results for OS. For the combined cohort, age and grade remained significantly associated with OS. Age > 40 years (HR, 2.06; 95% CI, 1.18-3.59; *p = .011*) was associated with worse OS. Patients with grade 2 astrocytoma had better OS than those with grade 3 disease (HR, 0.42; 95% CI, 0.21-0.83; *p = .012*). *CDKN2A*/*B* wild-type (HR, 1.60; 95% CI, 0.73-3.50; *p = .24*) and *BRCA2* wildtype (HR, 0.50; 95% CI, 0.22-1.13; *p = .09*) were not found to be statistically significant in the overall cohort.

**Table 3.** Summary of Multivariable Analysis of OS

Characteristic	HR (95% CI)	*p*
**All patients**		
Age: >40 years vs ≤40 years	2.06 (1.18-3.59)	**.011**
CNS WHO grade		
2 vs 3	0.42 (0.21-0.83)	**.012**
4 vs 3	1.70 (0.80-3.63)	.17
*CDKN2A/B*		
WT vs mutation	1.60 (0.73-3.50)	.24
Not tested vs mutation	1.90 (0.72-5.00)	.20
EOR		
GTR vs non-GTR	0.66 (0.34-1.26)	.21
Unknown vs non-GTR	0.48 (0.23-1.03)	.059
*BRCA2*		
WT vs mutation	0.50 (0.22-1.13)	.09
Not tested vs mutation	0.32 (0.09-1.15)	.08
**Institution #1 cohort**		
Age: >40 years vs ≤=40 years	1.67 (0.78-3.54)	.18
CNS WHO grade		
2 vs 3	0.67 (0.30-1.50)	.33
4 vs 3	2.75 (0.96-7.89)	.06
*CDKN2A/B*		
WT vs mutation	1.47 (0.51-4.25)	.47
Not tested vs mutation	2.49 (0.75-8.24)	.14
EOR		
GTR vs non-GTR	0.57 (0.25-1.30)	.18
Unknown vs non-GTR	0.53 (0.23-1.20)	.13
**Institution #2 cohort**		
Age: > 40 years vs ≤ 40 years	2.02 (0.78-5.28)	.15
CNS WHO grade		
2 vs 3	0.17 (0.04-0.85)	**.031**
4 vs 3	0.82 (0.27-2.47)	.73
*CDKN2A/B*: WT vs mutation	0.95 (0.25-3.58)	.93
EOR: GTR vs non-GTR	1.41 (0.43-4.58)	.57

Abbreviations: CNS WHO, Central Nervous System World Health Organization; EOR, extend of resection; GTR, gross total resection; WT, wild-type. Bold numbers present statistically significant values.

### Tumor Mutational Burden

TMB calculation requires the analysis of mutations in a large number genes (requiring a large NGS panel) and the information was only available for 39 patients. Of the 39 patients, 3 samples with TMB > 10 and 1 sample with missing value were excluded, leaving 35 samples. The median TMB was 4 for Institution #1 and 2 for Institution #2. No significant differences in TMB between the Institution #1 and Institution #2 cohorts were noted (*p* > .09 for all tests); thus, the evaluation of TMB and survival included all 35 patients.

**Figure 3:**
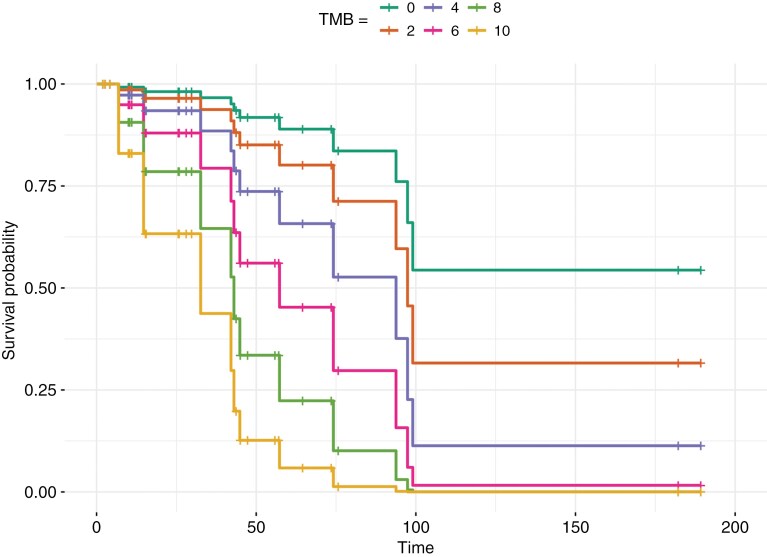
Predicted survival curves for different values of TMB. This plot shows the estimated survival probabilities over time for 6 strata based on the value of TMB. The *x*-axis represents survival time in months, and the *y*-axis represents the survival probability. Each curve corresponds to a specific TMB value. One patient had TMB = 0 (aqua color), 12 patients had TMB = 1 or 2 (orange color), 16 patients had TMB = 3 or 4 (lavender color), 2 patients had TMB = 5 or 6 (pink color), 3 patients had TMB = 7 or 8 (green color), and 1 patient had TMB = 9 or 10 (yellow color).

Patients with TMB ≤ 10 experienced worse OS with every unit increase in TMB (HR, 1.39; 95% CI, 1.02-1.86; *p* = .039; **Figure 3**). However, no significant association was observed between TMB and survival when including the 3 samples with TMB > 10 (HR, 0.98; 95% CI, 0.92-1.05; *p* = .64).

## Discussion

In this multi-institutional study, we observed some differences in the baseline patient characteristics between the 2 institutions, including age, race, and CNS WHO grade. A unifying explanation for this is lacking, but from our experience, these two institutions serve a different set of populations with differences in socioeconomic and health insurance status. The percent of non-White patients was 8% at Institution 1 and 20% at Institution 2. Taken together, the differences in socioeconomic status, health insurance status, and ethnic differences may have contributed to differential results.^13^

Results of both the univariate and multivariable assessments are consistent with those in prior studies demonstrating age to be an important prognostic factor in patients with *IDH*-mutant astrocytoma.^14^ A significant association between age and OS was observed in the combined cohort, however, this association was not statistically significant within a single institution, likely due to inadequate statistical power. In addition, the value of CNS WHO grading of *IDH*-mutant astrocytoma is controversial because of conflicting findings from studies regarding its relevance for risk stratification; the results of both the univariate and multivariable assessments in this retrospective study confirmed that grading is a valuable prognostic factor in patients with *IDH*-mutant astrocytoma even though the difference was observed only in between grade 2 and 3 in our study.^15,16^ This is consistent with the results of another study of similar size (258 patients with a median follow-up of 10.4 years) that also indicated that WHO grade was prognostic.^17^ Taken together, these results support the current practice of surveillance only after surgery for CNS WHO grade 2 *IDH*-mutant astrocytoma without high-risk features, such as age > 40 years, and consideration of adjuvant treatment in grade 3 or 4 tumors.^18,19^

In terms of mutations, a cross-sectional study demonstrated shorter OS in patients with low-grade gliomas with *BRCA1* or *BRCA2* mutations.^20^ In this retrospective study, *BRCA2* mutation was a prognostic factor in univariate analysis but not in multivariable analysis. Further studies are warranted to assess its role in risk stratification for *IDH*-mutant astrocytoma. Additionally, in this study, higher TMB suggested a significantly shorter survival time for samples with TMB ≤ 10. Scant literature on glioma has shown that higher TMB may be associated with shorter OS for diffuse gliomas.^21,22^ There are no set guidelines or definitions for TMB calculations and/or what constitutes high TMB in diffuse gliomas. Nonetheless, the results are consistent with prior studies showing that patients with *IDH*-mutant astrocytomas with mutations in MMR genes, which have a higher number of mutations, have shorter mOS.^8^

In the univariate analysis, increased Ki67 labeling (a surrogate immunohistochemical proliferation marker) was prognostic for *IDH*-mutant astrocytoma in Institution #1 cohort. We did not evaluate these measures in Institution #2 multivariable analysis because the data was not available. Prior studies uncovered the prognostic value of Ki67 index for gliomas, including low-grade tumors, but the exact criteria for risk stratification remain unclear.^15,23,24^

We acknowledge that previous studies demonstrated that EOR of *IDH*-mutant astrocytoma was associated with survival.^25–27^ mOS for the patients who underwent GTR in this series was longer than that in patients who underwent non-GTR procedures, but the difference was not statistically significant, possibly due to the low number of patients who underwent GTR (48 of the 241 patients) as defined by review of imaging studies by the neuroradiologists and limitations in the length of follow up in this study.

Prior studies have shown that homozygous deletion of *CDKN2A*/*B* is associated with poor survival in *IDH*-mutant astrocytoma.^28–30^ Similar to the EOR data here, mOS for all patients who had a homozygous deletion of *CDKN2A*/*B* was shorter than that in patients with wildtype *CDKN2A/B*, but the difference was not statistically significant. This is likely due to the limited sample size and the limited length of follow-up in this study. A recent study in 347 *IDH-*mutant patients found that hemizygous-deleted or homozygous-deleted *CDKN2A/B* are associated with statistically shortened mOS.^31^ A limitation of this study, in addition to its retrospective nature, was the different baseline patient characteristics between the two institutions, which highlights the challenge of having a uniform multi-institutional study cohort. However, a multi-institutional cohort provides a better representation of the general population of patients with *IDH*-mutant astrocytoma despite the study size being moderate. As a result, generalizations based on the TMB data were also limited. In addition, the median follow-up duration in this study (3.8 years) was shorter than that in some long-term clinical trials such as Radiation Therapy Oncology Group trial 9802 (9.0 years).^32^ We also did not include tumor location or neuroimaging features in this study, hence limiting the discussion on those aspects. Some strengths of this study include the extensive annotated data on molecular alterations from next-generation sequencing, the associated survival data, histological grade, and neuroimaging-based determination of the EOR.

In summary, this study supports that age and CNS WHO Grade are essential elements of risk stratification for *IDH*-mutant astrocytoma. Further studies are warranted to confirm the role of *BRCA2* mutations and TMB in the prognosis of this disease.

## Supplementary Material

vdaf088_suppl_Supplementary_Data

vdaf088_suppl_Supplementary_Tables_S1-S2

## Data Availability

The data supporting the findings of this study are available upon reasonable request.

## References

[1] Louis DN , Perry A, Wesseling P, et al The 2021 WHO classification of tumors of the central nervous system: a summary. Neuro Oncol. 2021;23(8):1231–1251. 34185076 10.1093/neuonc/noab106 PMC8328013

[2] Tesileanu CMS , Vallentgoed WR, French PJ, van den Bent MJ. Molecular markers related to patient outcome in patients with IDH-mutant astrocytomas grade 2 to 4: a systematic review. Eur J Cancer. 2022;175(1):214–223. 36152406 10.1016/j.ejca.2022.08.016

[3] Mellinghoff IK , van den Bent MJ, Blumenthal DT, et al; INDIGO Trial Investigators. Vorasidenib in IDH1- or IDH2-mutant low-grade glioma. N Engl J Med. 2023;389(7):589–601. 37272516 10.1056/NEJMoa2304194 PMC11445763

[4] Petersen JK , Boldt HB, Sørensen MD, et al Targeted next-generation sequencing of adult gliomas for retrospective prognostic evaluation and up-front diagnostics. Neuropathol Appl Neurobiol. 2021;47(1):108–126. 32696543 10.1111/nan.12645

[5] Shirahata M , Ono T, Stichel D, et al Novel, improved grading system(s) for IDH-mutant astrocytic gliomas. Acta Neuropathol. 2018;136(1):153–166. 29687258 10.1007/s00401-018-1849-4

[6] Wang K , Wang YY, Ma J, et al Prognostic value of MGMT promoter methylation and TP53 mutation in glioblastomas depends on IDH1 mutation. Asian Pac J Cancer Prev. 2014;15(24):10893–10898. 25605197 10.7314/apjcp.2014.15.24.10893

[7] Lee K , Kim SI, Kim EE, et al Genomic profiles of IDH-mutant gliomas: MYCN-amplified IDH-mutant astrocytoma had the worst prognosis. Sci Rep. 2023;13(1):6761. 37185778 10.1038/s41598-023-32153-y PMC10130138

[8] Richardson TE , Yokoda RT, Rashidipour O, et al Mismatch repair protein mutations in isocitrate dehydrogenase (IDH)-mutant astrocytoma and IDH-wild-type glioblastoma. Neurooncol. Adv. 2023;5(1):vdad085. 37554222 10.1093/noajnl/vdad085 PMC10406418

[9] Yang RR , Li KK, Zhang ZY, et al Mismatch repair proteins PMS2 and MLH1 can further refine molecular stratification of IDH-mutant lower grade astrocytomas. Clin Neurol Neurosurg. 2021;208(1):106882. 34428613 10.1016/j.clineuro.2021.106882

[10] Śledzińska P , Bebyn MG, Furtak J, Kowalewski J, Lewandowska MA. Prognostic and Predictive Biomarkers in Gliomas. Int J Mol Sci. 2021;22(19):10373. 34638714 10.3390/ijms221910373 PMC8508830

[11] Franceschi E , Tosoni A, Bartolini S, et al Histopathological grading affects survival in patients with IDH-mutant grade II and grade III diffuse gliomas. Eur J Cancer. 2020;137(1):10–17. 32721633 10.1016/j.ejca.2020.06.018

[12] Milbury CA , Creeden J, Yip WK, et al Clinical and analytical validation of FoundationOne®CDx, a comprehensive genomic profiling assay for solid tumors. PLoS One. 2022;17(3):e0264138. 35294956 10.1371/journal.pone.0264138 PMC8926248

[13] Hill, L. Health Coverage by Race and Ethnicity, 2010-2022. Kaiser Family Foundation. Updated Jan 11, 2024. https://www.kff.org/racial-equity-and-health-policy/issue-brief/health-coverage-by-race-and-ethnicity/. Accessed June 26, 2024.

[14] Ostrom QT , Shoaf ML, Cioffi G, et al National-level overall survival patterns for molecularly-defined diffuse glioma types in the United States. Neuro Oncol. 2023;25(4):799–807. 35994777 10.1093/neuonc/noac198 PMC10076944

[15] Brat DJ , Aldape K, Colman H, et al cIMPACT-NOW update 5: recommended grading criteria and terminologies for IDH-mutant astrocytomas. Acta Neuropathol. 2020;139(3):603–608. 31996992 10.1007/s00401-020-02127-9 PMC8443062

[16] Olar A , Wani KM, Alfaro-Munoz KD, et al IDH mutation status and role of WHO grade and mitotic index in overall survival in grade II-III diffuse gliomas. Acta Neuropathol. 2015;129(4):585–596. 25701198 10.1007/s00401-015-1398-z PMC4369189

[17] Weller M , Felsberg J, Hentschel B, et al Improved prognostic stratification of patients with isocitrate dehydrogenase-mutant astrocytoma. Acta Neuropathol. 2024;147(1):11. 38183430 10.1007/s00401-023-02662-1 PMC10771615

[18] Geurts M , van den Bent MJ. On high-risk, low-grade glioma: what distinguishes high from low? Cancer. 2019;125(2):174–176. 30512190 10.1002/cncr.31834 PMC6587541

[19] Miller JJ , Gonzalez Castro LN, McBrayer S, et al Isocitrate dehydrogenase (IDH) mutant gliomas: a Society for Neuro-Oncology (SNO) consensus review on diagnosis, management, and future directions. Neuro Oncol. 2023;25(1):4–25. 36239925 10.1093/neuonc/noac207 PMC9825337

[20] Meimand SE , Pour-Rashidi A, Shahrbabak MM, et al The prognostication potential of BRCA genes expression in gliomas: a genetic survival analysis study. World Neurosurg. 2022;157(1):e123–e128. 34607064 10.1016/j.wneu.2021.09.107

[21] Wang L , Ge J, Lan Y, et al Tumor mutational burden is associated with poor outcomes in diffuse glioma. BMC Cancer. 2020;20(1):213. 32164609 10.1186/s12885-020-6658-1 PMC7069200

[22] Yin W , Jiang X, Tan J, et al Development and validation of a tumor mutation burden-related immune prognostic model for lower-grade glioma. Front Oncol. 2020;10(1):1409. 32974146 10.3389/fonc.2020.01409 PMC7468526

[23] Chen WJ , He DS, Tang RX, Ren FH, Chen G. Ki-67 is a valuable prognostic factor in gliomas: evidence from a systematic review and meta-analysis. Asian Pac J Cancer Prev. 2015;16(2):411–420. 25684464 10.7314/apjcp.2015.16.2.411

[24] Fisher BJ , Naumova E, Leighton CC, et al Ki-67: a prognostic factor for low-grade glioma? Int J Radiat Oncol Biol Phys. 2002;52(4):996–1001. 11958894 10.1016/s0360-3016(01)02720-1

[25] Motomura K , Chalise L, Ohka F, et al Impact of the extent of resection on the survival of patients with grade II and III gliomas using awake brain mapping. J Neurooncol. 2021;153(2):361–372. 34009509 10.1007/s11060-021-03776-w

[26] Patel SH , Bansal AG, Young EB, et al Extent of surgical resection in lower-grade gliomas: Differential impact based on molecular subtype. AJNR Am J Neuroradiol. 2019;40(7):1149–1155. 31248860 10.3174/ajnr.A6102 PMC7048539

[27] Beiko J , Suki D, Hess KR, et al IDH1 mutant malignant astrocytomas are more amenable to surgical resection and have a survival benefit associated with maximal surgical resection. Neuro Oncol. 2014;16(1):81–91. 24305719 10.1093/neuonc/not159 PMC3870823

[28] Appay R , Dehais C, Maurage CA, et al; POLA Network. CDKN2A homozygous deletion is a strong adverse prognosis factor in diffuse malignant IDH-mutant gliomas. Neuro Oncol. 2019;21(12):1519–1528. 31832685 10.1093/neuonc/noz124 PMC7145561

[29] Yuile A , Satgunaseelan L, Wei JQ, et al CDKN2A/B Homozygous deletions in astrocytomas: a literature review. Curr Issues Mol Biol. 2023;45(7):5276–5292. 37504251 10.3390/cimb45070335 PMC10378679

[30] Wong QH , Li KK, Wang WW, et al Molecular landscape of IDH-mutant primary astrocytoma grade IV/glioblastomas [published correction appears in Mod Pathol. 2021 Jun;34(6):1231]. Mod Pathol. 2021;34(7):1245–1260. 33692446 10.1038/s41379-021-00778-x

[31] Hickman RA , Gedvilaite E, Ptashkin R, et al CDKN2A/B mutations and allele-specific alterations stratify survival outcomes in IDH-mutant astrocytomas. Acta Neuropathol. 2023;146(6):845–847. 37831210 10.1007/s00401-023-02639-0 PMC10628020

[32] Bell EH , Zhang P, Shaw EG, et al Comprehensive genomic analysis in NRG oncology/RTOG 9802: a phase III trial of radiation versus radiation plus procarbazine, lomustine (CCNU), and vincristine in high-risk low-grade glioma. J Clin Oncol. 2020;38(29):3407–3417. 32706640 10.1200/JCO.19.02983 PMC7527157

