# Supplementary material for: Molecular alterations in IDH-mutant astrocytoma: A multi-institutional retrospective study
Source: Neurooncol Adv. 2025 Apr 28;7(1):vdaf088. doi: 10.1093/noajnl/vdaf088 (PMC12199353; doi:10.1093/noajnl/vdaf088)
Supplement: vdaf088_suppl_Supplementary_Tables_S1-S2 [file vdaf088_suppl_supplementary_tables_s1-s2.docx]

**Supplemental Table 1. Summary of Molecular Alterations**

|  |  | ***n* (%)** | | |  |
| --- | --- | --- | --- | --- | --- |
| **Molecular Alterations** |  | **All Patients**  **(*N* = 241)** | **Institution #1 Cohort**  **(*N* = 185)** | **Institution #2 Cohort**  **(*N* = 56)** | ***P*** ^a^ |
| *CDKN2A/B** |  |  |  |  |  |
| Mutation |  | 26 (13) | 17 (12) | 9 (16) | .49 |
| WT |  | 168 (87) | 121 (88) | 47 (84) |  |
| Not tested |  | 47 | 47 | 0 |  |
| *AKT1* |  |  |  |  |  |
| Mutation |  | 1 (<1) | 0 | 1 (2) | .24 |
| WT |  | 237 (100) | 182 (100) | 55 (98) |  |
| Not tested |  | 3 | 3 | 0 |  |
| *AKT2* |  |  |  |  |  |
| Mutation |  | 1 (<1) | 0 | 1 (2) | .27 |
| WT |  | 210 (100) | 155 (100) | 55 (98) |  |
| Not tested |  | 30 | 30 | 0 |  |
| *ALK* |  |  |  |  |  |
| Mutation |  | 5 (2) | 3 (2) | 2 (4) | .34 |
| WT |  | 231 (98) | 177 (98) | 54 (96) |  |
| Not tested |  | 5 | 5 | 0 |  |
| *AR* |  |  |  |  |  |
| Mutation |  | 2 (1) | 2 (1) | 0 | 1.00 |
| WT |  | 224 (99) | 168 (99) | 56 (100) |  |
| Not tested |  | 15 | 15 | 0 |  |
| *ARID1A* |  |  |  |  |  |
| Mutation |  | 17 (8) | 10 (6) | 7 (13) | .16 |
| WT |  | 194 (92) | 145 (94) | 49 (88) |  |
| Not tested |  | 30 | 30 | 0 |  |
| *ATM* |  |  |  |  |  |
| Mutation |  | 10 (4) | 8 (4) | 2 (4) | 1.00 |
| WT |  | 226 (96) | 172 (96) | 54 (96) |  |
| Not tested |  | 5 | 5 | 0 |  |
| *ATR* |  |  |  |  |  |
| Mutation |  | 8 (4) | 6 (4) | 2 (4) | 1.00 |
| WT |  | 202 (96) | 148 (96) | 54 (96) |  |
| Not tested |  | 31 | 31 | 0 |  |
| *ATRX* |  |  |  |  |  |
| Mutation |  | 147 (70) | 104 (67) | 43 (77) | .23 |
| WT |  | 64 (30) | 51 (33) | 13 (23) |  |
| Not tested |  | 30 | 30 | 0 |  |
| *AXL* |  |  |  |  |  |
| Mutation |  | 3 (1) | 1 (1) | 2 (4) | .17 |
| WT |  | 208 (99) | 154 (99) | 54 (96) |  |
| Not tested |  | 30 | 30 | 0 |  |
| *BAP1* |  |  |  |  |  |
| Mutation |  | 1 (<1) | 1 (1) | 0 | 1.00 |
| WT |  | 222 (100) | 166 (99) | 56 (100) |  |
| Not tested |  | 18 | 18 | 0 |  |
| *BRAF* |  |  |  |  |  |
| Mutation |  | 6 (3) | 2 (1) | 4 (7) | **.029** |
| WT |  | 232 (97) | 180 (99) | 52 (93) |  |
| Not tested |  | 3 | 3 | 0 |  |
| *BRCA1* |  |  |  |  |  |
| Mutation |  | 8 (4) | 5 (3) | 3 (5) | .42 |
| WT |  | 215 (96) | 162 (97) | 53 (95) |  |
| Not tested |  | 18 | 18 | 0 |  |
| *BRCA2* |  |  |  |  |  |
| Mutation |  | 14 (6) | 7 (4) | 7 (13) | **.049** |
| WT |  | 209 (94) | 160 (96) | 49 (88) |  |
| Not tested |  | 18 | 18 | 0 |  |
| *CBL* |  |  |  |  |  |
| Mutation |  | 3 (1) | 1 (1) | 2 (4) | .16 |
| WT |  | 220 (99) | 166 (99) | 54 (96) |  |
| Not tested |  | 18 | 18 | 0 |  |
| *CCND1* |  |  |  |  |  |
| Mutation |  | 1 (<1) | 1 (1) | 0 | 1.00 |
| WT |  | 222 (100) | 166 (99) | 56 (100) |  |
| Not tested |  | 18 | 18 | 0 |  |
| *CCND2* |  |  |  |  |  |
| Mutation |  | 5 (2) | 3 (2) | 2 (4) | .61 |
| WT |  | 205 (98) | 151 (98) | 54 (96) |  |
| Not tested |  | 31 | 31 | 0 |  |
| *CCND3* |  |  |  |  |  |
| Mutation |  | 1 (<1) | 1 (1) | 0 | 1.00 |
| WT |  | 209 (100) | 153 (99) | 56 (100) |  |
| Not tested |  | 31 | 31 | 0 |  |
| *CCNE1* |  |  |  |  |  |
| Mutation |  | 2 (1) | 2 (1) | 0 | 1.00 |
| WT |  | 221 (99) | 165 (99) | 56 (100) |  |
| Not tested |  | 18 | 18 | 0 |  |
| *CDK2* |  |  |  |  |  |
| Mutation |  | 1 (1) | 1 (1) | 0 | 1.00 |
| WT |  | 153 (99) | 153 (99) | 0 |  |
| Not tested |  | 87 | 31 | 56 |  |
| *CDK4* |  |  |  |  |  |
| Mutation |  | 9 (4) | 7 (4) | 2 (4) | 1.00 |
| WT |  | 215 (96) | 161 (96) | 54 (96) |  |
| Not tested |  | 17 | 17 | 0 |  |
| *CDK6* |  |  |  |  |  |
| Mutation |  | 5 (2) | 2 (1) | 3 (5) | .10 |
| WT |  | 219 (98) | 166 (99) | 53 (95) |  |
| Not tested |  | 17 | 17 | 0 |  |
| *CDKN1B* |  |  |  |  |  |
| Mutation |  | 4 (2) | 3 (2) | 1 (2) | 1.00 |
| WT |  | 206 (98) | 151 (98) | 55 (98) |  |
| Not tested |  | 31 | 31 | 0 |  |
| *CDKN2A* |  |  |  |  |  |
| Mutation |  | 13 (6) | 4 (2) | 9 (16) | **<.001** |
| WT |  | 223 (94) | 176 (98) | 47 (84) |  |
| Not tested |  | 5 | 5 | 0 |  |
| *CDKN2B* |  |  |  |  |  |
| Mutation |  | 4 (2) | 2 (1) | 2 (4) | .29 |
| WT |  | 207 (98) | 153 (99) | 54 (96) |  |
| Not tested |  | 30 | 30 | 0 |  |
| *CHEK1* |  |  |  |  |  |
| Mutation |  | 3 (1) | 3 (2) | 0 | .57 |
| WT |  | 208 (99) | 152 (98) | 56 (100) |  |
| Not tested |  | 30 | 30 | 0 |  |
| *CHEK2* |  |  |  |  |  |
| Mutation |  | 1 (<1) | 1 (1) | 0 | 1.00 |
| WT |  | 223 (100) | 167 (99) | 56 (100) |  |
| Not tested |  | 17 | 17 | 0 |  |
| *CREBBP* |  |  |  |  |  |
| Mutation |  | 13 (6) | 10 (6) | 3 (5) | 1.00 |
| WT |  | 197 (94) | 144 (94) | 53 (95) |  |
| Not tested |  | 31 | 31 | 0 |  |
| *CSF1R* |  |  |  |  |  |
| Mutation |  | 2 (1) | 0 | 2 (4) | .056 |
| WT |  | 234 (99) | 180 (100) | 54 (96) |  |
| Not tested |  | 5 | 5 | 0 |  |
| *DDR2* |  |  |  |  |  |
| Mutation |  | 1 (<1) | 1 (1) | 0 | 1.00 |
| WT |  | 222 (100) | 166 (99) | 56 (100) |  |
| Not tested |  | 18 | 18 | 0 |  |
| *EGFR* |  |  |  |  |  |
| Mutation |  | 8 (3) | 5 (3) | 3 (5) | .40 |
| WT |  | 228 (97) | 175 (97) | 53 (95) |  |
| Not tested |  | 5 | 5 | 0 |  |
| *ERBB2* |  |  |  |  |  |
| Mutation |  | 4 (2) | 3 (2) | 1 (2) | 1.00 |
| WT |  | 232 (98) | 177 (98) | 55 (98) |  |
| Not tested |  | 5 | 5 | 0 |  |
| *ERBB3* |  |  |  |  |  |
| Mutation |  | 1 (<1) | 1 (1) | 0 | 1.00 |
| WT |  | 222 (100) | 166 (99) | 56 (100) |  |
| Not tested |  | 18 | 18 | 0 |  |
| *ERBB4* |  |  |  |  |  |
| Mutation |  | 2 (1) | 0 | 2 (4) | .056 |
| WT |  | 234 (99) | 180 (100) | 54 (96) |  |
| Not tested |  | 5 | 5 | 0 |  |
| *EZH2* |  |  |  |  |  |
| Mutation |  | 3 (1) | 1 (1) | 2 (4) | .14 |
| WT |  | 236 (99) | 182 (99) | 54 (96) |  |
| Not tested |  | 2 | 2 | 0 |  |
| *FANCA* |  |  |  |  |  |
| Mutation |  | 2 (1) | 1 (1) | 1 (2) | .42 |
| WT |  | 235 (99) | 180 (99) | 55 (98) |  |
| Not tested |  | 4 | 4 | 0 |  |
| *FGF3* |  |  |  |  |  |
| Mutation |  | 2 (1) | 0 | 2 (4) | .055 |
| WT |  | 235 (99) | 181 (100) | 54 (96) |  |
| Not tested |  | 4 | 4 | 0 |  |
| *FGFR3* |  |  |  |  |  |
| Mutation |  | 2 (1) | 0 | 2 (4) | .055 |
| WT |  | 235 (99) | 181 (100) | 54 (96) |  |
| Not tested |  | 4 | 4 | 0 |  |
| *FGFR4* |  |  |  |  |  |
| Mutation |  | 2 (1) | 0 | 2 (4) | .055 |
| WT |  | 235 (99) | 181 (100) | 54 (96) |  |
| Not tested |  | 4 | 4 | 0 |  |
| *FOXL2* |  |  |  |  |  |
| Mutation |  | 1 (<1) | 0 | 1 (2) | .24 |
| WT |  | 236 (100) | 181 (100) | 55 (98) |  |
| Not tested |  | 4 | 4 | 0 |  |
| *GATA2* |  |  |  |  |  |
| Mutation |  | 1 (<1) | 0 | 1 (2) | .24 |
| WT |  | 236 (100) | 181 (100) | 55 (98) |  |
| Not tested |  | 4 | 4 | 0 |  |
| *GNA11* |  |  |  |  |  |
| Mutation |  | 2 (1) | 0 | 2 (4) | .055 |
| WT |  | 235 (99) | 181 (100) | 54 (96) |  |
| Not tested |  | 4 | 4 | 0 |  |
| *GNAS* |  |  |  |  |  |
| Mutation |  | 4 (2) | 0 | 4 (7) | **.003** |
| WT |  | 233 (98) | 181 (100) | 52 (93) |  |
| Not tested |  | 4 | 4 | 0 |  |
| *HNF1A* |  |  |  |  |  |
| Mutation |  | 2 (1) | 0 | 2 (4) | .055 |
| WT |  | 235 (99) | 181 (100) | 54 (96) |  |
| Not tested |  | 4 | 4 | 0 |  |
| *HRAS* |  |  |  |  |  |
| Mutation |  | 1 (<1) | 0 | 1 (2) | .24 |
| WT |  | 236 (100) | 181 (100) | 55 (98) |  |
| Not tested |  | 4 | 4 | 0 |  |
| *IDH1* |  |  |  |  |  |
| Mutation |  | 225 (95) | 170 (93) | 55 (98) | .31 |
| WT |  | 13 (5) | 12 (7) | 1 (2) |  |
| Not tested |  | 3 | 3 | 0 |  |
| *IDH2* |  |  |  |  |  |
| Mutation |  | 4 (2) | 1 (1) | 3 (5) | **.042** |
| WT |  | 234 (98) | 181 (99) | 53 (95) |  |
| Not tested |  | 3 | 3 | 0 |  |
| *IGF1R* |  |  |  |  |  |
| Mutation |  | 1 (<1) | 0 | 1 (2) | .24 |
| WT |  | 237 (100) | 182 (100) | 55 (98) |  |
| Not tested |  | 3 | 3 | 0 |  |
| *JAK1* |  |  |  |  |  |
| Mutation |  | 3 (1) | 1 (1) | 2 (4) | .14 |
| WT |  | 234 (99) | 180 (99) | 54 (96) |  |
| Not tested |  | 4 | 4 | 0 |  |
| *JAK2* |  |  |  |  |  |
| Mutation |  | 1 (<1) | 0 | 1 (2) | .24 |
| WT |  | 237 (100) | 182 (100) | 55 (98) |  |
| Not tested |  | 3 | 3 | 0 |  |
| *JAK3* |  |  |  |  |  |
| Mutation |  | 2 (1) | 1 (1) | 1 (2) | .42 |
| WT |  | 236 (99) | 181 (99) | 55 (98) |  |
| Not tested |  | 3 | 3 | 0 |  |
| *KDR* |  |  |  |  |  |
| Mutation |  | 5 (2) | 0 | 5 (9) | **<.001** |
| WT |  | 232 (98) | 181 (100) | 51 (91) |  |
| Not tested |  | 4 | 4 | 0 |  |
| *KIT* |  |  |  |  |  |
| Mutation |  | 6 (3) | 0 | 6 (11) | **<.001** |
| WT |  | 231 (97) | 181 (100) | 50 (89) |  |
| Not tested |  | 4 | 4 | 0 |  |
| *KRAS* |  |  |  |  |  |
| Mutation |  | 2 (1) | 0 | 2 (4) | .055 |
| WT |  | 235 (99) | 181 (100) | 54 (96) |  |
| Not tested |  | 4 | 4 | 0 |  |
| *MAP2K2* |  |  |  |  |  |
| Mutation |  | 1 (<1) | 0 | 1 (2) | .24 |
| WT |  | 236 (100) | 181 (100) | 55 (98) |  |
| Not tested |  | 4 | 4 | 0 |  |
| *MDM4* |  |  |  |  |  |
| Mutation |  | 1 (<1) | 0 | 1 (2) | .24 |
| WT |  | 236 (100) | 181 (100) | 55 (98) |  |
| Not tested |  | 4 | 4 | 0 |  |
| *MED12* |  |  |  |  |  |
| Mutation |  | 5 (2) | 0 | 5 (9) | **<.001** |
| WT |  | 232 (98) | 181 (100) | 51 (91) |  |
| Not tested |  | 4 | 4 | 0 |  |
| *MET* |  |  |  |  |  |
| Mutation |  | 2 (1) | 0 | 2 (4) | .055 |
| WT |  | 236 (99) | 182 (100) | 54 (96) |  |
| Not tested |  | 3 | 3 | 0 |  |
| *MLH1* |  |  |  |  |  |
| Mutation |  | 1 (<1) | 1 (1) | 0 | 1.00 |
| WT |  | 236 (100) | 180 (99) | 56 (100) |  |
| Not tested |  | 4 | 4 | 0 |  |
| *MRE11A* |  |  |  |  |  |
| Mutation |  | 1 (<1) | 0 | 1 (2) | .24 |
| WT |  | 234 (100) | 179 (100) | 55 (98) |  |
| Not tested |  | 6 | 6 | 0 |  |
| *MSH2* |  |  |  |  |  |
| Mutation |  | 1 (<1) | 0 | 1 (2) | .24 |
| WT |  | 236 (100) | 181 (100) | 55 (98) |  |
| Not tested |  | 4 | 4 | 0 |  |
| *MSH6* |  |  |  |  |  |
| Mutation |  | 3 (1) | 0 | 3 (5) | **.012** |
| WT |  | 235 (99) | 182 (100) | 53 (95) |  |
| Not tested |  | 3 | 3 | 0 |  |
| *MTOR* |  |  |  |  |  |
| Mutation |  | 2 (1) | 1 (1) | 1 (2) | .42 |
| WT |  | 235 (99) | 180 (99) | 55 (98) |  |
| Not tested |  | 4 | 4 | 0 |  |
| *MYC* |  |  |  |  |  |
| Mutation |  | 2 (1) | 0 | 2 (4) | .055 |
| WT |  | 235 (99) | 181 (100) | 54 (96) |  |
| Not tested |  | 4 | 4 | 0 |  |
| *MYCL* |  |  |  |  |  |
| Mutation |  | 3 (1) | 0 | 3 (5) | **.013** |
| WT |  | 234 (99) | 181 (100) | 53 (95) |  |
| Not tested |  | 4 | 4 | 0 |  |
| *MYCN* |  |  |  |  |  |
| Mutation |  | 5 (2) | 0 | 5 (9) | **<.001** |
| WT |  | 232 (98) | 181 (100) | 51 (91) |  |
| Not tested |  | 4 | 4 | 0 |  |
| *MYD88* |  |  |  |  |  |
| Mutation |  | 1 (<1) | 0 | 1 (2) | .23 |
| WT |  | 238 (100) | 183 (100) | 55 (98) |  |
| Not tested |  | 2 | 2 | 0 |  |
| *NF1* |  |  |  |  |  |
| Mutation |  | 7 (3) | 2 (1) | 5 (9) | **.009** |
| WT |  | 231 (97) | 180 (99) | 51 (91) |  |
| Not tested |  | 3 | 3 | 0 |  |
| *NF2* |  |  |  |  |  |
| Mutation |  | 4 (2) | 0 | 4 (7) | **.003** |
| WT |  | 233 (98) | 181 (100) | 52 (93) |  |
| Not tested |  | 4 | 4 | 0 |  |
| *NOTCH1* |  |  |  |  |  |
| Mutation |  | 7 (3) | 0 | 7 (13) | **<.001** |
| WT |  | 230 (97) | 181 (100) | 49 (88) |  |
| Not tested |  | 4 | 4 | 0 |  |
| *NOTCH2* |  |  |  |  |  |
| Mutation |  | 1 (<1) | 0 | 1 (2) | .24 |
| WT |  | 236 (100) | 181 (100) | 55 (98) |  |
| Not tested |  | 4 | 4 | 0 |  |
| *NOTCH3* |  |  |  |  |  |
| Mutation |  | 3 (1) | 0 | 3 (5) | **.013** |
| WT |  | 234 (99) | 181 (100) | 53 (95) |  |
| Not tested |  | 4 | 4 | 0 |  |
| *NRAS* |  |  |  |  |  |
| Mutation |  | 1 (<1) | 0 | 1 (2) | .24 |
| WT |  | 237 (100) | 182 (100) | 55 (98) |  |
| Not tested |  | 3 | 3 | 0 |  |
| *NTRK1* |  |  |  |  |  |
| Mutation |  | 3 (1) | 0 | 3 (5) | **.012** |
| WT |  | 235 (99) | 182 (100) | 53 (95) |  |
| Not tested |  | 3 | 3 | 0 |  |
| *NTRK3* |  |  |  |  |  |
| Mutation |  | 1 (<1) | 0 | 1 (2) | .24 |
| WT |  | 237 (100) | 182 (100) | 55 (98) |  |
| Not tested |  | 3 | 3 | 0 |  |
| *PDGFRA* |  |  |  |  |  |
| Mutation |  | 8 (3) | 0 | 8 (14) | **<.001** |
| WT |  | 229 (97) | 181 (100) | 48 (86) |  |
| Not tested |  | 4 | 4 | 0 |  |
| *PDGFRB* |  |  |  |  |  |
| Mutation |  | 5 (2) | 2 (1) | 3 (5) | .09 |
| WT |  | 233 (98) | 180 (99) | 53 (95) |  |
| Not tested |  | 3 | 3 | 0 |  |
| *PIK3CA* |  |  |  |  |  |
| Mutation |  | 6 (3) | 0 | 6 (11) | **<.001** |
| WT |  | 231 (97) | 181 (100) | 50 (89) |  |
| Not tested |  | 4 | 4 | 0 |  |
| *PIK3R1* |  |  |  |  |  |
| Mutation |  | 5 (2) | 1 (1) | 4 (7) | **.011** |
| WT |  | 234 (98) | 182 (99) | 52 (93) |  |
| Not tested |  | 2 | 2 | 0 |  |
| *POLE* |  |  |  |  |  |
| Mutation |  | 1 (<1) | 0 | 1 (2) | .24 |
| WT |  | 236 (100) | 181 (100) | 55 (98) |  |
| Not tested |  | 4 | 4 | 0 |  |
| *PTCH1* |  |  |  |  |  |
| Mutation |  | 3 (1) | 0 | 3 (5) | **.013** |
| WT |  | 234 (99) | 181 (100) | 53 (95) |  |
| Not tested |  | 4 | 4 | 0 |  |
| *PTEN* |  |  |  |  |  |
| Mutation |  | 1 (<1) | 0 | 1 (2) | .24 |
| WT |  | 236 (100) | 181 (100) | 55 (98) |  |
| Not tested |  | 4 | 4 | 0 |  |
| *PTPN11* |  |  |  |  |  |
| Mutation |  | 2 (1) | 2 (1) | 0 | 1.00 |
| WT |  | 237 (99) | 181 (99) | 56 (100) |  |
| Not tested |  | 2 | 2 | 0 |  |
| *RAD50* |  |  |  |  |  |
| Mutation |  | 2 (1) | 0 | 2 (4) | .055 |
| WT |  | 235 (99) | 181 (100) | 54 (96) |  |
| Not tested |  | 4 | 4 | 0 |  |
| *RAD51* |  |  |  |  |  |
| Mutation |  | 2 (1) | 0 | 2 (4) | .055 |
| WT |  | 235 (99) | 181 (100) | 54 (96) |  |
| Not tested |  | 4 | 4 | 0 |  |
| *RAF1* |  |  |  |  |  |
| Mutation |  | 1 (<1) | 0 | 1 (2) | .24 |
| WT |  | 236 (100) | 181 (100) | 55 (98) |  |
| Not tested |  | 4 | 4 | 0 |  |
| *RB1* |  |  |  |  |  |
| Mutation |  | 1 (<1) | 1 (1) | 0 | 1.00 |
| WT |  | 236 (100) | 180 (99) | 56 (100) |  |
| Not tested |  | 4 | 4 | 0 |  |
| *RET* |  |  |  |  |  |
| Mutation |  | 2 (1) | 1 (1) | 1 (2) | .41 |
| WT |  | 237 (99) | 182 (99) | 55 (98) |  |
| Not tested |  | 2 | 2 | 0 |  |
| *RICTOR* |  |  |  |  |  |
| Mutation |  | 2 (1) | 0 | 2 (4) | .055 |
| WT |  | 235 (99) | 181 (100) | 54 (96) |  |
| Not tested |  | 4 | 4 | 0 |  |
| *RNF43* |  |  |  |  |  |
| Mutation |  | 1 (<1) | 1 (1) | 0 | 1.00 |
| WT |  | 237 (100) | 181 (99) | 56 (100) |  |
| Not tested |  | 3 | 3 | 0 |  |
| *ROS1* |  |  |  |  |  |
| Mutation |  | 2 (1) | 0 | 2 (4) | .055 |
| WT |  | 235 (100) | 181 (100) | 54 (98) |  |
| Not tested |  | 5 | 4 | 1 |  |
| *SETD2* |  |  |  |  |  |
| Mutation |  | 3 (1) | 0 | 3 (5) | **.012** |
| WT |  | 235 (99) | 182 (100) | 53 (95) |  |
| Not tested |  | 3 | 3 | 0 |  |
| *SF3B1* |  |  |  |  |  |
| Mutation |  | 1 (<1) | 0 | 1 (2) | .24 |
| WT |  | 237 (100) | 182 (100) | 55 (98) |  |
| Not tested |  | 3 | 3 | 0 |  |
| *SMAD4* |  |  |  |  |  |
| Mutation |  | 1 (<1) | 0 | 1 (2) | .23 |
| WT |  | 238 (100) | 183 (100) | 55 (98) |  |
| Not tested |  | 2 | 2 | 0 |  |
| *SMARCA4* |  |  |  |  |  |
| Mutation |  | 2 (1) | 0 | 2 (4) | .055 |
| WT |  | 235 (99) | 181 (100) | 54 (96) |  |
| Not tested |  | 4 | 4 | 0 |  |
| *SMO* |  |  |  |  |  |
| Mutation |  | 4 (2) | 0 | 4 (7) | **.003** |
| WT |  | 235 (98) | 183 (100) | 52 (93) |  |
| Not tested |  | 2 | 2 | 0 |  |
| *STK11* |  |  |  |  |  |
| Mutation |  | 1 (<1) | 0 | 1 (2) | .24 |
| WT |  | 236 (100) | 181 (100) | 55 (98) |  |
| Not tested |  | 4 | 4 | 0 |  |
| *TERT* |  |  |  |  |  |
| Mutation |  | 3 (1) | 1 (1) | 2 (4) | .14 |
| WT |  | 234 (99) | 180 (99) | 54 (96) |  |
| Not tested |  | 4 | 4 | 0 |  |
| *TOP1* |  |  |  |  |  |
| Mutation |  | 1 (<1) | 0 | 1 (2) | .24 |
| WT |  | 236 (100) | 181 (100) | 55 (98) |  |
| Not tested |  | 4 | 4 | 0 |  |
| *TP53* |  |  |  |  |  |
| Mutation |  | 215 (91) | 162 (90) | 53 (95) | .30 |
| WT |  | 22 (9) | 19 (10) | 3 (5) |  |
| Not tested |  | 4 | 4 | 0 |  |
| *TSC1* |  |  |  |  |  |
| Mutation |  | 9 (4) | 6 (4) | 3 (5) | .69 |
| WT |  | 214 (96) | 161 (96) | 53 (95) |  |
| Not tested |  | 18 | 18 | 0 |  |
| *TSC2* |  |  |  |  |  |
| Mutation |  | 14 (6) | 10 (6) | 4 (7) | .75 |
| WT |  | 209 (94) | 157 (94) | 52 (93) |  |
| Not tested |  | 18 | 18 | 0 |  |
| *XPO1* |  |  |  |  |  |
| Mutation |  | 2 (1) | 2 (1) | 0 | 1.00 |
| WT |  | 221 (99) | 165 (99) | 56 (100) |  |
| Not tested |  | 18 | 18 | 0 |  |

^a^ Fisher’s exact test or its generalization; ^b^ Wilcoxon rank-sum test. *copy number loss; all molecular alterations are otherwise single nucleotide variants

**Supplemental Table 2. Summary of Univariate Analysis Results for PFS**

| **Characteristic** |  | **Median PFS Time, Months**  **(95% CI)** | **PFS Rate, %**  **(YR1:YR5:YR10: Last)** | ***P*** | **HR (95% CI)** | ***P*** |
| --- | --- | --- | --- | --- | --- | --- |
| **All Patients** | | | | | | |
| Overall |  | 59.7 (45.8-72.4) | 88:50:22:3 |  |  |  |
| Age |  |  |  |  |  |  |
| ≤40 years |  | 61.5 (48.0-75.4) | 89:51:24:4 | .15 | *ref* |  |
| >40 years |  | 43.7 (29.8-88.9) | 86:46:9:0 |  | 1.36 (0.90-2.05) | .15 |
| CNS WHO Grade |  |  |  |  |  |  |
| 2 |  | 75.4 (48.0-101.1) | 89:60:28:9 | .14 | 0.73 (0.47-1.14) | .16 |
| 3 |  | 55.3 (39.3-69.9) | 89:47:13:0 |  | *ref* |  |
| 4 |  | 43.7 (32.0-59.7) | 87:36:18:5 |  | 1.09 (0.70-1.70) | .71 |

**Abbreviations:** PFS, progression free survival; YR1, year 1 after initial diagnosis; YR5, year 5 after initial diagnosis; YR10, year 10 after initial diagnosis; Last, as of last censor date; CNS WHO, Central Nervous System World Health Organization
